# MRI-Induced Heating of Coils for Microscopic Magnetic Stimulation at 1.5 Tesla: An Initial Study

**DOI:** 10.3389/fnhum.2020.00053

**Published:** 2020-03-13

**Authors:** Giorgio Bonmassar, Peter Serano

**Affiliations:** ^1^Athinoula A. Martinos Center, Massachusetts General Hospital, Harvard Medical School, Charlestown, MA, United States; ^2^ANSYS Inc., Canonsburg, PA, United States

**Keywords:** finite elements method, RF-induced heating, specif absorption rate, bioheat equation, deep brain stimulation implant

## Abstract

**Purpose:**

Deep brain stimulation (DBS) has proved to be effective in the treatment of movement disorders. However, the direct contact between the metal contacts of the DBS electrode and the brain can cause RF heating in magnetic resonance imaging (MRI) scanning, due to an increase of local specific absorption rate (SAR). Recently, micro coils (μMS) have demonstrated excitation of neuronal tissue through the electromagnetic induction both *in vitro* and *in vivo* experiments. In contrast to electrical stimulation, in μMS, there is no direct contact between the metal and the biological tissue.

**Methods:**

We compared the heating of a μMS coil with a control case of a metal wire. The heating was induced by RF fields in a 1.5 T MRI head birdcage coil (often used for imaging patients with implants) at 64 MHz, and normalized results to 3.2 W/kg whole head average SAR.

**Results:**

The μMS coil or wire implants were placed inside an anatomically accurate head saline-gel filled phantom inserted in the RF coil, and we observed approximately 1°C initial temperature rise at the μMS coil, while the wire exhibited a 10°C temperature rise in the proximity of the exposed end. The numerical simulations showed a 32-times increase of local SAR induced at the tips of the metal wire compared to the μMS.

**Conclusion:**

In this work, we show with measurements and electromagnetic numerical simulations that the RF-induced increase in local SAR and induced heating during MRI scanning can be greatly reduced by using magnetic stimulation with the proposed μMS technology.

## Introduction

Active implanted medical devices (AIMDs) based on electrical stimulation such as pacemakers (Gold et al., 2017), spinal cord stimulators (Patel et al., 2017), and cardioverter-defibrillators (Proclemer et al., 2001) have become a standard therapeutic choice to restore healthy neural activity in a wide range of medical conditions (D’Haese et al., 2010). In the brain, deep brain stimulation (DBS) uses electrical stimulation for the treatment of several medically refractory brain disorders, including essential tremor, Parkinson’s disease, major depression, dystonia, Tourette syndrome, chronic pain, and obsessive-compulsive disorder (Lefaucheur et al., 2004; Walter and Vitek, 2004; Montgomery and Gale, 2008; Machado et al., 2009, 2012; Plow et al., 2009; Holtzheimer and Mayberg, 2011; Vitek et al., 2011; Machado and Baker, 2012; Plow et al., 2012; Graat et al., 2017; Clair et al., 2018). Despite their remarkable success, significant limitations are still curtailing the use of AIMDs. For instance, as none of AIMDs’ electrodes are currently completely magnetic resonance imaging (MRI) safe, a full exploration of their clinical utility is limited as there is a lost opportunity to bridge the gap between functional and structural MRI and neurophysiology. MRI is a gold-standard diagnostic tool due to its non-invasive nature and excellent soft-tissue contrast but has safety concerns in patients with implants. Brain hemorrhages have been reported in two patients with bilateral DBS implants due to the excessive heating of electrodes during the scan: the first patient suffered a permanent hemiparalysis (Henderson et al., 2005), and the second experienced temporary dystonia (Spiegel et al., 2003). In these patients, radiofrequency (RF) waves generated with MRI that interact (“antenna-effect”) (Serano et al., 2015) with the crude metal wires in the leads to induce currents, which is the leading cause of local specific absorption rate (SAR) and heating increase (Angelone et al., 2010; Golestanirad et al., 2019). Unfortunately, increased local SAR and potentially excessive temperature increase near the DBS electrodes can lead to tissue necrosis (Bassen et al., 2006). As a result, patients with brain implants are often rejected from MRI examinations as they are not eligible (Erhardt et al., 2018). Furthermore, the use of follow-up MRI at many centers is precluded, even for critical clinical assessments to diagnose comorbidities such as bleeds near the electrodes, stroke, cancer, etc. (Zrinzo et al., 2011). Despite recent changes in the labeling of Medtronic’s MRI DBS systems, which now allow the use of RF body coils, the landscape has mainly remained curtailed as the allowable RF field (i.e., *B*_1_,*rms*^+^ < 2μT) and the main field (i.e., B_0_ = 1.5T) have continued to be extremely restrictive, precluding full use of MRI.

**FIGURE 1:**
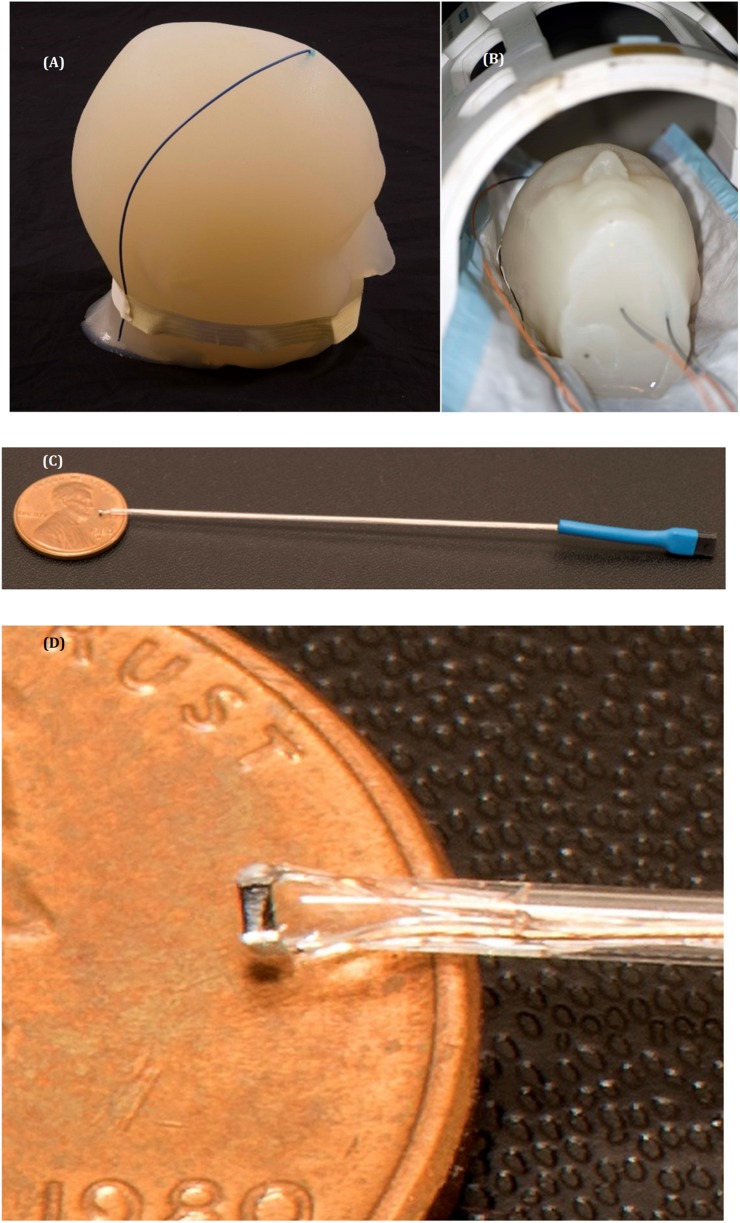
Temperature measurements setup. **(A)** The gel phantom with the metal wire implanted. **(B)** The gel phantom with implanted μMS coil and the fluoroptic temperature sensors inside the Tx/Rx birdcage head coil in a 1.5 T system. **(C,D)** Images of the μMS implant and a detailed view of the μMS coil.

Using microscopic magnetic stimulation (μMS), we successfully elicited neuronal activation in an *in vitro* retinal preparation (Serano et al., 2015), and we similarly showed activation of neuronal circuitry at the system level *in vivo* (Bonmassar et al., 2012). A recent paper (Golestanirad et al., 2018) computed the electric field induced by the μMS coil adjacent to neuronal tissue in deep brain areas of the brainstem and combined these electromagnetic simulation results (Bonmassar et al., 2010) with axon cable models to investigate μMS orientation-specific properties (i.e., ability to excite neurons with axons perpendicular but not parallel to the coil), which were then validated in animal models. In contrast to electrical stimulation, which requires an exposed conductive lead tip in contact with the tissue, μMS (Figure 1) has a conformal dielectric coating or insulation all around the implant that significantly attenuates the flow of the antenna-effect induced currents between the metal and the biological tissue, thus reducing the induced heating. Additionally, the μMS coil provides an increased inductance in the lead due to the turns in the coil, which previous research has shown experimentally to decrease induced heating near the implant (Bottomley et al., 2010).

## Materials and Methods

We performed studies with both MRI temperature measurements (physical) and electromagnetic simulations (numerical), to study in a 1.5 T MRI head birdcage coil (often used for imaging patients with implants) the RF-induced heating of an μMS coil vs. a microwire. A saline-gel filled anthropomorphic head phantom was also implanted with a wire (Mattei et al., 2007) as a control condition to test that the temperature measurements and simulations were performed correctly. We studied physically and numerically the RF-induced heating of an implanted μMS coil (a 21-turns spiral wounding with a circular cross-section of 400 μm in diameter) since this coil was used in all the *in vitro* experiments in Serano et al. (2015), and *in vivo* experiments in Bonmassar et al. (2012) and Golestanirad et al. (2018). The 21-turns coil was originally selected since, at that time, it was the smallest off-the-shelf coil available that provided the maximum amount of inductance, thus capable of producing the maximum amount of magnetic energy for neurostimulation. The μMS coil had thin electrical insulation everywhere to insulate from electricity/current resulting from the applied voltage, while the wire was similarly insulated everywhere except for the open end (i.e., tip) inside the head, which was exposed to model a lead with an electrode-type contact. All geometrical dimensions and material properties of the coils (MRI and μMS) and leads were identical for the physical and numerical studies (Supplementary Table S1).

### MRI Temperature Measurements and Image Quality (Physical)

**FIGURE 2:**
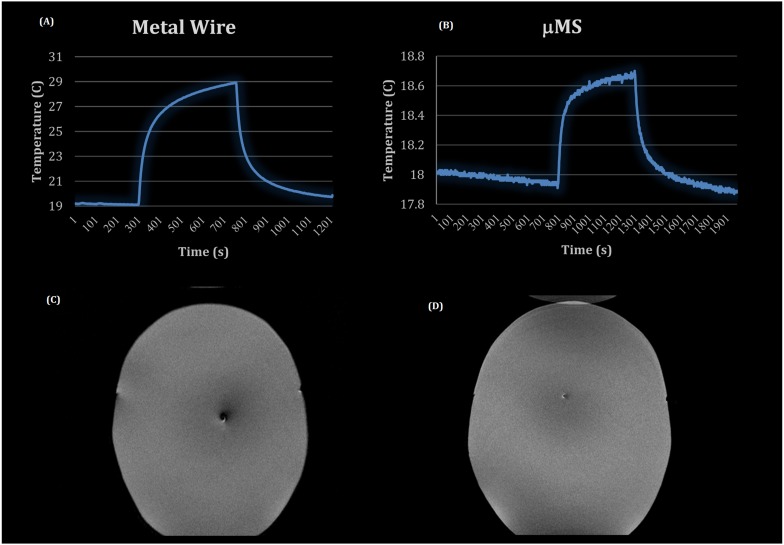
MRI experiments: temperature profiles for the metal wire **(A)** and the μMS coil **(B)**, T2 images of the CHEMA phantom with **(C)** the metal wire implanted and **(D)** with the μMS coil implanted.

The temperature measurements were performed with the fiber optic temperature probe positioned within 1 mm^3^ of the μMS coil or exposed end of the wire for reproducibility. The μMS coil or wire implants were placed inside the phantom while the phantom was inserted in the RF Coil field of view. The actual MRI heating experiments (Figure 2) were performed with a gel-filled phantom and an implanted wire or μMS. A turbo-spin echo sequence (TR = 6s, FA = 120°, 1 slice) was used on a 1.5 T Magnetom Avanto Tim system (Siemens, Erlangen, Germany) to deliver a SAR = 3.2 W/kg to the phantom using a quadrature birdcage transmit–receive coil used at the A.A. Martinos Center for clinical head imaging patients with implants (see Supplementary Material for more info on the temperature measurements and micro coil construction). Image quality was assessed by using a common clinical sequence [T2 turbo spin echo (TSE), TR = 4s, TE = 104 ms, 19 slices 3 mm in-plane resolution].

### Simulations (Numerical)

**FIGURE 3:**
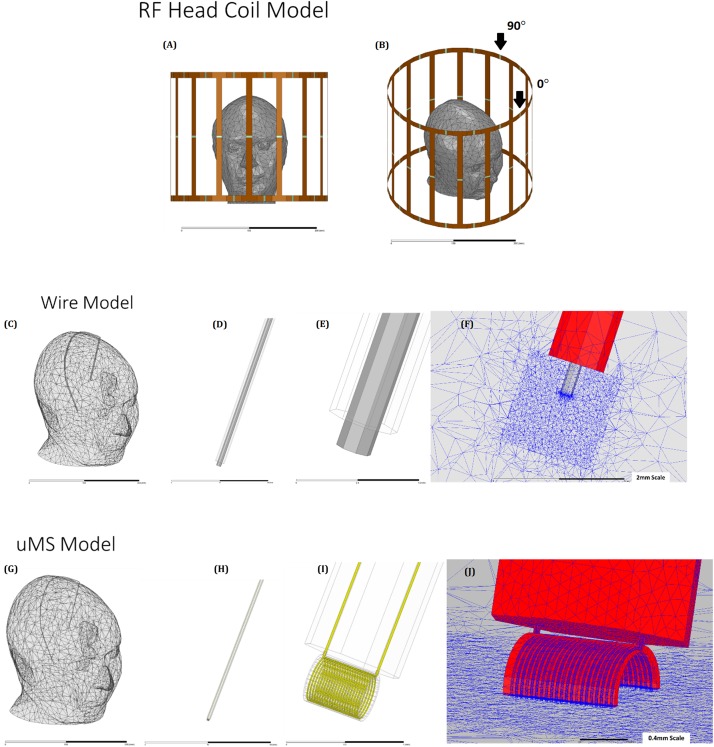
The geometrical model: **(A)** and **(B)** head model (Plow et al., 2009) placed in a 1.5 T shielded birdcage coil. **(C)** Wire implant in the head model, showing outer insulation **(D)**, exposed wire tip **(E)**, and details of the mesh **(F)** head model **(G)** with the leads **(H)**, μMS coil **(I)**, and details of the mesh **(J)**.

The numerical simulations were based on the finite element method (FEM) and compute the electric and magnetic fields throughout the model. Electromagnetic simulations were implemented in ANSYS HFSS (v19.2, ANSYS Inc., Canonsburg, PA, United States). A numerical model of a shielded high-pass birdcage head coil (see Supplementary Table S1) was implemented and tuned to the 1.5 T with a Larmor frequency of 64 MHz (Figures 3A,B). The numerical head coil matched the coil used in the measurements (Bonmassar et al., 2013), and the geometry consisted of two end rings, which were connected through 16 rungs. The material used for modeling the rings of the head coil as well as the shield was copper. Two ports were feeding along the end-ring of the birdcage coil to create a quadrature excitation with a 0° in-phase and with a 90° polar angle shift (Supplementary Figure S1). A set of lumped capacitors (75 pF) distributed at the end-ring tuned the head coil numerically, and a 50 Ω impedance matching was achieved by introducing at each port a 50 Ω internal resistance in series with an ideal voltage source and a single capacitor (77 pF). The ensemble of the head coil and head model (Makris et al., 2008) were enclosed in an air-filled cylinder with a “radiation” absorbing boundary condition (ABC) to ensure adequate absorption of radiated energy from the coil to the outer boundary of the model. In the wire model, a wire was implanted in the head model (Figure 3) with dimensions listed in Supplementary Table S1. The microcoil was a thin film inductor (Figure 1C) with a total of 21 turns, and the traces were: 25 μm thick, 25 μm wide, and 450 μm long attached to a 30 cm two-wire extension (Rezai et al., 2002), Figure 3G shows only the straight part of the implant, which was then connected to an extension (Figure 3F). Maximum mesh element sizes were enforced on various geometries to adequately capture the induced current in the μMS coil with a maximum mesh element size of 1 μm on the μMS coil geometry and 10 μm on the wire lead tip. In each simulation, the final lead mesh was visually inspected to assure that microscopic features of the wire lead and μMS coil were properly represented, specifically to assure that the closely wound loops of the μMS coil were not bridged during the meshing process. Electromagnetic fields were calculated using HFSS and were used to evaluate the SAR and the temperature increase due to the implant (Figures 3A,B). In all simulations, the amplitude of the two voltage sources was adjusted to produce a whole-body SAR value of 3.2 W/kg in the head—the “normal operating” power level for clinical MRI systems. Simulations were considered converged after a maximum change of 1% of the peak E-field magnitude anywhere inside the model.

#### SAR Simulations

Simulations were performed to determine the values of local power deposited in the phantom in the case of metal wire and μMS coil. The SAR averaged over 1 g of tissue (SAR_1__g_). SAR_1__g_ was adopted to estimate the power in the phantom and was calculated according to the IEEE STD P1528.4 recommendation, using a built-in module that estimates the local SAR at each mesh edge ($S\,A\,R_{1\,g}=\frac{\sigma \,||E^{2}||}{2\,\rho }$ whereσ is the material conductivity [S], ||E|| is the complex magnitude of the electric field [V/m], and ρ is the density of the material [kg/m^3^]). The SAR averaged over 1 g (SAR_1__g_) was calculated by averaging the local SAR over a volume of 1 g of mass that surrounded each mesh point, and the volume was calculated using the material’s mass density. Each simulation took approximately 2 h on a Dell PowerEdge R730 system running with 16 processing cores and utilized approximately 120 GB RAM.

#### Temperature Simulations

All of the temperature simulations solved the following heat partial differential equation in solids, which corresponds to the differential form of heat equation:

(1) $$\rho \,C_{\text{p}}\,\frac{\partial T}{\partial t}=\nabla \cdot \left(k_{\text{T}}\,\nabla T\right)+\int \int \int_{\emptyset }\frac{\sigma \,{||E||}^{2}}{\rho }\,dx\,dy\,dz$$

where ρ = 1000 [kg/m^3^] is the mass density,*C*_p_ = 4150[J/(kg K)] is the specific heat capacity at constant pressure (1 bar),*T* [K] is the absolute temperature, *k*_T_ = 0.42 [W/(m K)] is the thermal conductivity, and ∅ = 1cm^3^ is the volume of 1 g of gel in the point where *T* is estimated. The heat equation in solids (Eq. 1) was solved in terms of T using the transient thermal solver of ANSYS Mechanical (v19.2, ANSYS Inc., Canonsburg, PA, United States), and the Dirichlet boundary conditions were set on the external surfaces of the head phantom to heat transfer by conduction only with *T* = 18^o^C. The right term of the equation is the driving heat source, which was the SAR_1__g_ calculated with the HFSS (see above) with the power normalization to 3.2 W/kg averaged whole head SAR to match experiments. The geometry consisted of the CHEMA head model with one tissue (Angelone et al., 2006). The solution was calculated in a Cartesian 3D coordinate system and consisted of temperature values*T*. The thermal solver is dynamically linked with the electromagnetic solver using ANSYS Workbench (v19.2, ANSYS Inc., Canonsburg, PA, United States). Transient thermal simulations were performed for all cases over a total time of 500 s using a 0.5 s minimum time step. A maximum mesh size of 0.1 mm was applied to the μMS and wire lead to ensure the capture of fine details of the implants. Each simulation took approximately 1 h on a Dell PowerEdge R730 system running with 16 processing cores and utilized approximately 46 GB RAM. Simulations converged when the norm of the N-R load reached fluctuations within a tolerance of 10^–3^ and when the L2 norm reached a minimum reference value of 10^–6^.

## Results

### MRI Temperature Measurement and Image Quality

Two MRI safety experiments included using a high-power SAR TSE sequence in the CHEMA phantom included: a control case of a metal wire (Figure 2A) and the implant with the μMS coil (Figure 2B). In particular, a total of 7 min with a TSE sequence that was adjusted to provide slightly over 100% SAR generated two different temperature waveforms for the μMS coil and the copper wire. The implant with the μMS coil produced just below 1°C heating, which is the heating induced by a 3.2 W/kg whole head SAR in a phantom. In particular, the metal wire heated with a peak of 10°C above a baseline room temperature of 19°C. The μMS coil heating peak was instead only 0.8°C. Slight cooling of the 18°C baseline was observed given that the phantom was at a warmer temperature than the room after the wire heating MRI scan.

The μMS coil and the control case of a metal wire MR T2 images (MEMPRAGE) were compared and showed a larger artifact size for the metallic wire (Figure 2C) over the microcoil (Figure 2D). This simple qualitative study was performed only to show that the current induced in the coil does not produce excessive artifacts for the most common clinical sequence (T2).

### Numerical Simulations

**FIGURE 4:**
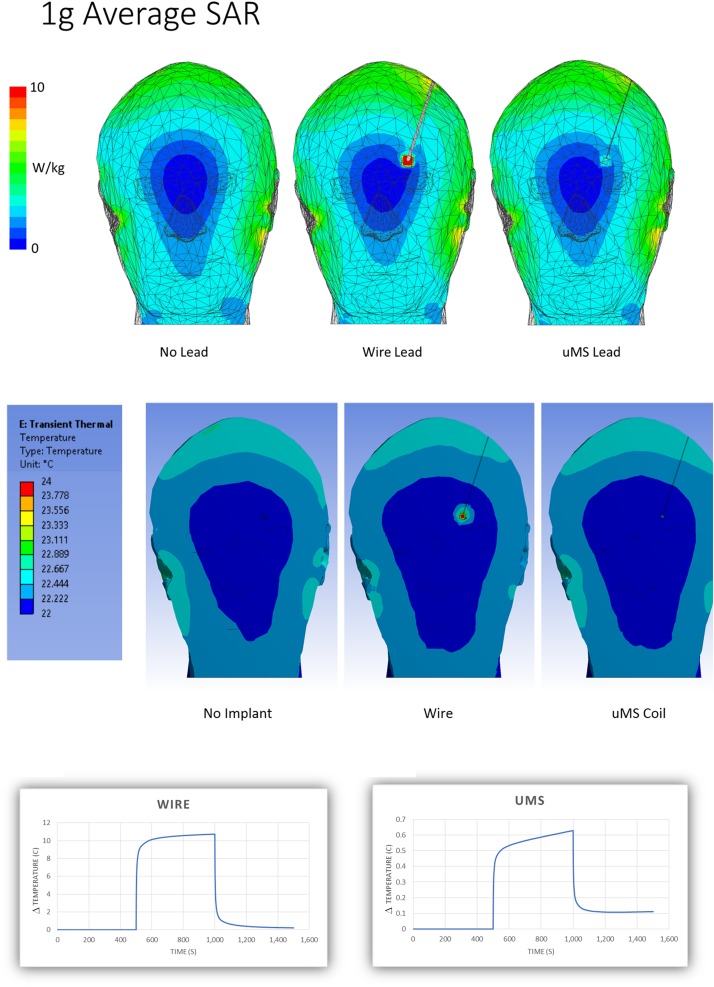
Simulation results. Estimation of the spatial distribution of the magnitude of the peak 1 g-SAR **(top)** and peak temperature **(middle)**. **(Bottom)** Estimation of the temperature “changes” ΔT profiles for the metal wire **(left)** and μMS **(right)** cases.

In this work, we show with electromagnetic numerical simulations that the RF-induced large local SAR peak during MRI scanning can be reduced by 32 times using magnetic stimulation with a microcoil (μMS), which justified the significant reduction observed in tissue heating near the lead tip. The top row of Figure 4 shows the 1 g avg. SAR in the phantom for the no-lead (left), the metal wire lead (middle), and μMS coil (right). The 1-g averaged SAR simulations showed an increase in a maximum at the tip of the wire of 32.15 W/kg while the μMS coil exhibited only a 2.07 W/kg peak or a 15.5 × reduction. A similar tendency was present also in the local SAR (not shown) with an increase a maximum at the tip of the wire of 16.55 10^6^ W/kg, while the μMS coil exhibited only 0.52 10^6^ W/kg peak or a 31.8 × reduction. In particular, the J decrease inside the μMS implanted lead is due to the increase of inductance (i.e., RF-choke effect), while the J decrease in the proximity of the implant is due to the presence of the implant conformal insulation (i.e., fewer currents escape the implant).

The numerical simulations predicted a 10°C rise for the metal wire vs. 0.6°C for the μMS coil, which was well within the usual accuracy considered acceptable for this type of study (Serano et al., 2015). The simulations did not show any hot spot for the μMS coil, which was not possible to ascertain directly from the actual temperature measurements. Please see Supplementary Table S2 for further results of numerical simulations with the μMS coil with different lead trajectories.

Supplementary Table S3 presents the uncertainty table that studied the sensitivity of the results concerning design and simulation parameters. Please see Supplementary Material for details and the methodology used for the uncertainty table.

## Discussion

This study shows that the antenna effect (Angelone et al., 2010) and, consequently, tissue heating during MRI can be reduced by using microscopic magnetic stimulation or μMS instead of traditional electrical stimulation, both with actual thermal measurements and numerical simulations.

In a 1.5 T MRI, an implant can generate changes in temperature of 20^o^C or more in a saline solution phantom (Mattei et al., 2008). When an implant is immersed in a conductor and an incident RF field, the resulting proximal induced heating will depend on orientation and position (Neufeld et al., 2009). The thermal measurements were performed using fluoroptic temperature sensors (Sharan et al., 2003; Baker et al., 2004, 2006; Bhidayasiri et al., 2005; Gray et al., 2005; Mattei et al., 2007) to measure the RF-induced heating in the proximity of a microcoil and a wire during MRI. The thermal measurements were performed for the control case (i.e., copper wire) and the proposed μMS implant under the same electric field distribution inside the homogenous head phantom (Figure 3). The microcoil (μMS) that was studied here with electromagnetic and Pennes bioheat equation simulations and with MRI thermal experiments was the same coil used in the *in vitro* (Serano et al., 2015) and *in vivo* experiments (Bonmassar et al., 2012; Golestanirad et al., 2018) that extensively showed efficacy in eliciting neural stimulation.

Since this is only an initial study on a proof of concept prototype, we used a simple wire as a control condition, while a commercial DBS implant has multiple coiled conductors and various materials that affect the electromagnetic properties. In the MRI safety community, wires are an accepted control condition with no pretext of representing a real implant (Bottomley et al., 2010). Instead, wires are often tested as a control condition to show that T-measurements were appropriately performed (Mattei et al., 2007, 2008), and heating at the tip of the wire can be reliably detected. Also, the limitations of the study were that we did not study the case of bilateral lead implantation, and we only studied the 64 MHz RF excitation case of a 1.5 T scanner. Furthermore, both measurements and simulations did not model the exposure conditions in a patient with an implant (Fraix et al., 2010). A complete ISO TS 10974 industry-standard guidelines for implant testing of the safety of the proposed μMS implant is lacking, and further MRI safety analysis is necessary (ISO/TS 10974,2012; Cabot et al., 2013) before this technology is translated into the clinics. Further simulations on a finalized lead design with models that include all possible implant pathways and measurements with RF injection in the implant (Muthalaly et al., 2018; Brown et al., 2019) to simulate the exposure scenarios inside the scanner, as well as heating tests, will be required on the final implant. These tests are required by the FDA to ensure the worst-case scenario sufficiently meets the requirements for patient safety. A potential way to reduce the hefty measurement costs and the considerable computational load to comply with the TS 10974 guidelines is to adopt the transfer function (TF) (Park et al., 2007) estimation of the μMS implant. The TF allows computing the electric field in a point of interest, for instance, the electrode that determines the local heating, by convolving the TF (recently extended to wireless estimation; Tokaya et al., 2018) with the tangent component of the electric field distribution along the trajectory of the implant.

The numerical simulations were based on FEM (Serano et al., 2015) and provided information on the electromagnetic fields, SAR rate, and temperature. The FEM solver allows for a variable density of mesh elements, which enables the use of 1 μm sized mesh elements near the μMS coil and 1 cm sized mesh elements in the head. This variable mesh density creates computationally, efficient models that can be feasibly solved. In particular, this variable mesh allowed for a high geometrical modeling accuracy at the wire tip, where the highest electric field was observed (Guy, 1975; Mohsin et al., 2008; Neufeld et al., 2009). The high geometrical accuracy was particularly needed to model the microcoil, which is a rod of 400 μm in diameter and 21 turns 25 μm wide looped around the rod. While simulations performed with competing solvers such as the finite differences time domain (FDTD) would require much larger resources, such as CUDA cards with a memory of several terabytes, and computing times an order of magnitude or greater compared to the FEM solution. Preliminary validation of the accuracy of the FEM simulations was performed by comparing them with *in vitro* temperature measurements in a gel-filled head phantom, which is known as CHEMA, and that was previously used in many other similar MRI/safety studies (Angelone et al., 2006, 2010; Poulsen et al., 2016; Atefi et al., 2018). Further validations of the numerical simulations, such as the rotating B1 field generated by the head coil and the SAR, are presented in the Supplementary Material.

## Conclusion

μMS coils have the potential to become the electrode of choice in stimulation devices such as pacemakers and deep brain stimulators as well as many other FDA class III devices [e.g., “devices that sustain or support life are implanted, or present potential high risk of illness or injury.” (FDA, 2019)]. The ability to deliver the neuronal stimulation needed for therapeutic usage without physical contact with metal will confer to these devices: greater biocompatibility, enhanced MRI-safety, greater stimulation specificity, and focality, and protection against harmful charge accumulation. The development of such novel technology could result in significant benefits to patients that suffer from some medically refractory pathological conditions such as epilepsy, major depression, and migraine. Due to recent advancements in micro-machining technologies, it is possible to utilize inductors (or coils) constructed on the sub-millimeter scale to elicit neurostimulation via magnetic fields. FEM modeling allowed a more precise analysis of the relationship between SAR induced in the head and implants. The comparison was made with a metal wire that was introduced as a control condition to show that the temperature measurements were performed correctly (Mattei et al., 2007), and wires are sometimes used as a simplified model to study implant heating (Neufeld et al., 2009), but DBS implants contain multiple coiled wires instead. These results suggest that the copper wire implant did indeed show a remarkable local SAR peak near the tip of the wire, while the μMS coil exhibited an over 30-times decrease of local SAR. A similar trend was present for the 1 g averaged SAR (32 vs. 2 W/kg). While differences in SAR are not linearly mapped to temperature, the temperature measurements are in accordance with the SAR numerical estimation by also demonstrating a significant decrease in temperature, with the μMS coil lead producing less than 10x the temperature rise of the wire lead. Similar temperature reductions were predicted by the numerical simulations, which did not show any hot spots for the μMS case anywhere in the entire geometry. Finally, it should be noted that this is only an initial study on a proof of concept prototype, which is not a complete DBS implant; thus, the work does not meet the ISO TS 10974 industry-standard guidelines for active implant testing. However, the results shown suggest the proposed μMS based implant design may allow safe access to the diagnostic benefits of MRI to an increased number of patients with active implants.

## Data Availability Statement

The raw data supporting the conclusions of this article will be made available by the authors, without undue reservation, to any qualified researcher.

## Author Contributions

GB and PS conceived of the presented idea. GB developed the theory and performed the MRI experiments. PS performed the computations. GB encouraged PS to investigate the validation of the numerical simulations and supervised the findings of this work. All authors discussed the results and contributed to the final manuscript.

## Conflict of Interest

PS was employed by company ANSYS Inc. The remaining authors declare that the research was conducted in the absence of any commercial or financial relationships that could be construed as a potential conflict of interest.
