# Supplementary material for: MRI-Induced Heating of Coils for Microscopic Magnetic Stimulation at 1.5 Tesla: An Initial Study
Source: Front Hum Neurosci. 2020 Mar 13;14:53. doi: 10.3389/fnhum.2020.00053 (PMC7082860; doi:10.3389/fnhum.2020.00053)
Supplement: Supplementary file 1 [file Data_Sheet_1.docx]

**SUPPLEMENTARY INFORMATION**

MRI-induced Heating of Coils for Microscopic Magnetic Stimulation at 1.5 Tesla: an initial study.

Giorgio Bonmassar^1,3^ and Peter Serano^1,2^

^1^ Athinoula A. Martinos Center, Massachusetts General Hospital, Charlestown, MA, USA.

^2^ ANSYS Inc., Canonsburg, PA, USA

^3^ To whom correspondence should be addressed at Athinoula A. Martinos Center, Massachusetts General Hospital, Harvard Medical School, Building 149, 13^th^ Street, Charlestown, MA 02129, Tel. (617) 726-0962. Email: [giorgio.bonmassar@mgh.harvard.edu](mailto:giorgio.bonmassar@mgh.harvard.edu)

# **TABLE OF CONTENTS**

**SUPPLEMENTARY NOTES**

**MRI Temperature measurements (continued)**

**Wire-routing**

**Micro-coil Construction**

**Quadrature Coil Operation**

**Further Verification of SAR Modeling**

**Head Coil S-Parameters**

**Lead Path Variation**

**Uncertainty Analysis**

**SUPPLEMENTARY REFERENCES**

**SUPPLEMENTARY TABLE S1-3**

**SUPPLEMENTARY FIGURES S1-4**

**SUPPLEMENTARY NOTES**

**MRI Temperature measurements (continued)**

For comparison, we also measured temperature changes in the phantom with no implant and with a 16 AWG wire (Alpha wire Co, Elizabeth, NJ) for conventional testing. Leads were implanted into anthropomorphic head phantoms (**Fig. 1**) made of agarose-doped saline solution that mimicked the electrical and thermal properties of biological tissues ($\mathbf{Table S1}$), as described by Angelone et al. [1] (**Fig. 1B**). The fiber optic temperature sensors (Osensa Innovations Corp., Burnaby, Canada), with an accuracy of 0.25°C, were placed at the proximity (less than 1mm) albeit not in contact with the implant tip [2] and used to record the temperature in the phantom under several conditions: no leads, µMS leads, and a copper wire. RF energy was delivered to the phantom at First Level Controlled Operating Mode for 500 seconds or approximately 8 minutes when the temperatures plateaued.

**Wire-routing.**

Both the wire and µMS extension routing were not following the standard trajectory of a DBS implant in an actual patient given the difficulties of implanting in an agar-agar gel phantom, due to its brittle mechanical properties. The routing was a limitation of the study. However, the numerical simulations were accurately following this same trajectory for validation.

**Micro-coil Construction.**

In order to ensure high stimulation efficacy, all microcoils were constructed to keep overall resistances below 5Ω and inductances below 150nH (4263B, Keysight Technologies, Santa Rosa, CA). A multilayer inductor (ELJ-RFR10JFB, Panasonic Electronic Devices Corporation of America, Knoxville, TN), was attached by soldering to two 34-AWG copper wires (Philmore Mfg., Rockford, IL). In order to insulate the tissue from the voltage applied to the terminals and to protect against moisture, the micro-coils were brush coated with a Teflon Enamel, which offered high dielectric strength. The microscopic stimulator was mounted on a 23 AWG glass shaft and the two wires inserted in the shaft/hub of the needle and connected to a standard DIP connector.

**Quadrature Coil Operation.**

**Fig. S1** demonstrates the rotation of the H field vectors vs. phase of the input excitation in the head coil when loaded with our head model. The Figure shows four H-field vector plots distributions with four distinct phases of the input excitation (relative to the ϕ=0º driving port). The phases selected for validation of the circularly polarized and field homogeneity validation were: 0º, 90º, 180º, 270º.

**Further Verification of SAR Modeling.**

The 10g Average SAR was computed inside the head and was found (**Fig. S2**) to be less than 10 W/kg as in accordance with IEC 60601 in relation to the input power level which was set to the Normal Operation Mode (3.2W/kg WB Head SAR).

**Head Coil S-Parameters.**

S-Parameters were calculated with a 32MHz sweep centered at 64 MHz. The results show (**Fig. S3**) that the numerical head coil was properly tuned, matched, and decoupled quadrature RF coil system. Asymmetric loading of the coil due to the ellipsoidal shape of the head phantom can be observed with S11 matched to -14.9dB and S22 matched to -19.6dB.

**Lead Path Variation**

Additional lead path variations were generated by rotating the lead at the point where the lead enters the head. The results (**Table S2**) show that the temperature did not significantly change due to the change in the lead path (**Fig. S4**). While this analysis is certainly not as though as the ISO TS-10974 standard, it demonstrates in these cases that the worst-case temperature variation on the lead path was presented in the main text. A cooling up to 0.6° occurs as the implant shifts into a new trajectory, indicating that the results presented in **Fig. 4** were the worst case scenario.

**Uncertainty Table.**

A simulation study to assess the uncertainty of design and simulation parameters was performed (**Table S3**). The parameters studied were selected such that they could be considered independent. The methods used were based on the work of Neufeld et al. *[3]*. To determine the impact of the contribution of an individual parameter to the total uncertainty of the simulations, the first two simulations were run for each parameter by assigning two different values (“Val1” and “Val2”) to each parameter studied. The first value (“Val1”) was the one used for the simulations shown in Fig. 3, whereas the modified value (“Val2”) was set to a realistic value that could occur due to either design choice or manufacturing tolerance. Assuming linear dependence of the measurement values on the varying parameter, a sensitivity factor was determined for each parameter by calculating the percent error difference between the two evaluation results and then dividing by the absolute value of the change in the parameter value. The individual uncertainty contribution was then calculated by multiplying the sensitivity and the standard deviation of the parameter uncertainty. The standard deviations were small for parameters such as the implant length, which can be accurately determined, and large for parameters such as the conductivity. The analysis confirmed a high sensitivity – and relative high uncertainty – to the lead thickness and phantom conductivity. Lower SAR values resulted in a 12 mm vs. 24 mm lead diameter of leads, which is consistent with the notion that insulation characteristics strongly affect the antenna behavior [4]. Finally, the simulations showed that phantom conductivity plays a fundamental role in the RF-induced currents [5], with a low uncertainty coefficient (see Table S1). The uncertainty analysis performed showed that the dielectric constant of the insulator in the leads could significantly affect 10g-avg SAR.

**SUPPLEMENTARY REFERENCES**

1. Angelone, L.M., et al., *On the effect of resistive EEG electrodes and leads during 7 T MRI: simulation and temperature measurement studies.* Magn Reson Imaging, 2006. **24**(6): p. 801-12.

2. Mattei, E., et al., *Temperature and SAR measurement errors in the evaluation of metallic linear structures heating during MRI using fluoroptic probes.* Phys Med Biol, 2007. **52**(6): p. 1633-46.

3. Neufeld, E., et al., *Measurement, simulation and uncertainty assessment of implant heating during MRI.* Phys Med Biol, 2009. **54**(13): p. 4151-69.

4. Guy, A., *Biophysics-energy absorption and distribution.* AGARD Lecture Series, Radiation Hazards (Non-ionizing Radiations--Biological Effects and Safety Considerations, 1975. **78**.

5. Merilampi, S.L., et al., *The Effect of Conductive Ink Layer Thickness on the Functioning of Printed UHF RFID Antennas.* Proceedings of the IEEE, 2010. **98**(9): p. 1610-1619.

6. Serano, P., et al., *A novel brain stimulation technology provides compatibility with MRI.* Sci Rep, 2015. **5**: p. 9805.

**SUPPLEMENTARY TABLES**

| Geometry | Dimension |
| --- | --- |
| Head Coil Diameter | 352 mm |
| Head Coil Length | 292 mm |
| Head Coil Shield Diameter | 390 mm |
| Head Coil Shield Length | 360 mm |
| Head Coil and Shield Thickness | 0.1 mm |
| Head Coil Ring/Rung Width | 13 mm |
| Wire/μMS Lead Length | 30 cm |
| Wire/μMS Lead Diameter | 0.45 mm |
| Wire Conductor Diameter | 0.2 mm |
| μMS Conductor Diameter | 0.02 mm |
| μMS Coil Diameter | 0.4 mm |
| μMS Coil Length | 0.5 mm |
| μMS Coil Parylene C Thickness | 0.01 mm |
| Material | **Value** |
| Copper Conductivity (All Conductors) | 5.8 10^7^ S/m |
| Parylene C Dielectric Constant | 2.1 |
| Parylene C Tangent Loss | 0.001 |
| Phantom Conductivity | 0.47 S/m |
| Phantom Permittivity | 80 |
| Phantom Thermal Diffusivity | 1.3∙10^-7^ m/s^2^ |
| Phantom Specific Heat Capacity | 4150 J/(kg∙C) |
| Phantom Density | 1200 kg/m^3^ |

# **Table S1:** *(Top) Dimensions of the numerical model. Coil dimensions. (Bottom) Electrical and thermal properties used for the simulations.*

| Rotation [deg] | Temperature [C] |
| --- | --- |
| -15 | 22.6 |
| -12 | 22.7 |
| -9 | 22.7 |
| -6 | 22.9 |
| -3 | 23.1 |
| 0 | 23.2 |

**Table S2:** *Further µMS heating FEM simulations to test solution stability over the implant pathway. Five different path variations were generated by rotating the lead with a 3º step showing a MAX temperature decrease of 0.6º C.*

| Parameter | Quantity Evaluated | Value 1 | Value 2 | Result 1 | Result  2 | Sensitivity Factor [%/mm] | Std. Dev | Uncertainty [%] |
| --- | --- | --- | --- | --- | --- | --- | --- | --- |
| Lead Diameter [mm] | 10g Average SAR [W/kg] | 0.012 | 0.024 | 0.71 | 0.67 | 4.695 | 0.1 | 0.47 |
| Lead Conductivity [S/m] | 10g Average SAR [W/kg] | 5.8 107 | 4.0 106 | 0.71 | 0.70 | 0.000 | 0.1 | 0.00 |
| Insulation Diameter [mm] | 10g Average SAR [W/kg] | 0.45 | 0.9 | 0.71 | 0.84 | 0.407 | 0.1 | 0.04 |
| Insulation Dielectric Constant [-] | 10g Average SAR [W/kg] | 2.1 | 4.2 | 0.71 | 0.69 | 0.013 | 2 | 0.03 |
| Head Phantom Conductivity [S/m] | 10g Average SAR [W/kg] | 0.47 | 0.6 | 0.71 | 0.80 | 1.155 | 0.04 | 0.05 |
| Head Phantom Dielectric [-] | 10g Average SAR [W/kg] | 80 | 66.34 | 0.71 | 0.75 | 0.004 | 2 | 0.01 |
| Lead Length [cm] | 10g Average SAR [W/kg] | 30 | 20 | 0.71 | 0.62 | 0.013 | 2 | 0.03 |
| Position of Load in Coil X [mm] | Erms, Incident [V/m] | 0 | 10 | 45.6 | 57.8 | 0.027 | 1.15 | 0.03 |
| Position of Load in Coil Y [mm] | Erms, Incident [V/m] | 0 | 10 | 45.6 | 49.9 | 0.009 | 1.15 | 0.01 |
| Position of Load in Coil Z [mm] | Erms, Incident [V/m] | 0 | 10 | 45.6 | 39.9 | 0.012 | 1.15 | 0.01 |
| Lead Position in Head X [mm] | Erms, Incident [V/m] | 0 | 1 | 45.6 | 44.3 | 0.029 | 0.58 | 0.02 |
| Lead Position in Head Y [mm] | Erms, Incident [V/m] | 0 | 1 | 45.6 | 45.4 | 0.005 | 0.58 | 0.00 |
| Lead Position in Head Z [mm] | Erms, Incident [V/m] | 0 | 1 | 45.6 | 46.6 | 0.021 | 0.58 | 0.01 |

**Table S3: Uncertainty analysis.** *The methods used were based on the work of Neufeld et al. [3]. To evaluate the uncertainty of the quantities of interest derived by the simulations (i.e., 10 g-avg. SAR or the magnitude of incident electric field ‘‘ERMS’’) Two simulations were run for each parameter by assigning two different values (‘‘Val 1’’ and ‘‘Val 2’’) to each parameter studied. The first value (‘‘Val 1’’) was the one used for the simulations shown in* ***Fig. 4****, whereas the modified value (‘‘Val 2’’) was set to a realistic value that could occur due to either design choice or manufacturing tolerance. The results obtained for each value (‘‘Result 1’’ and ‘‘Result 2’’, respectively) were used to evaluate the sensitivity factor of the quantity evaluated (1 g-avg. SAR or magnitude of incident electric field ‘‘ERMS’’). The standard deviation (‘‘Std. Dev. ’’) was derived from the literature [6].*

**SUPPLEMENTARY FIGURES**


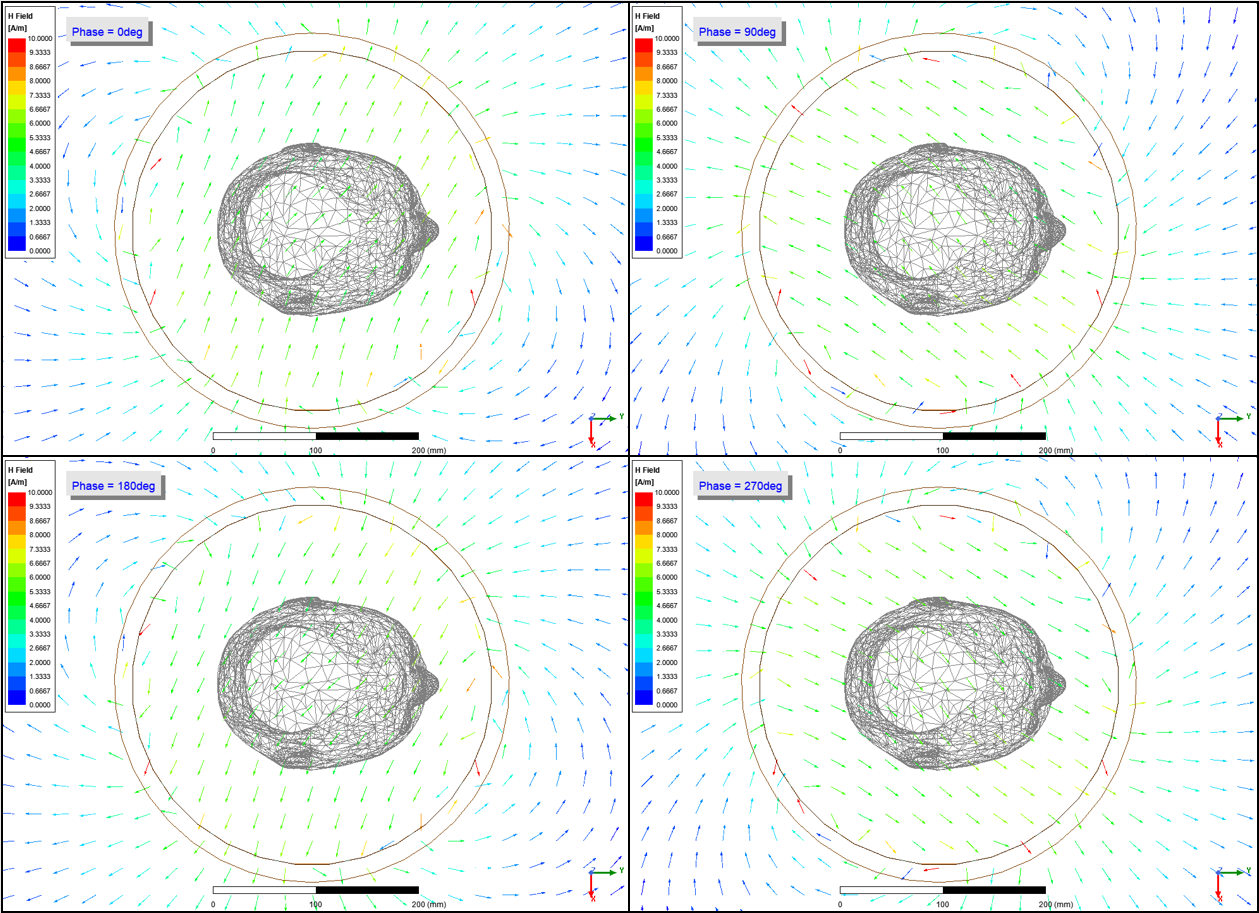


**Figure S1**: *Verification of Circularly Polarized (Quadrature) Coil Operation.*


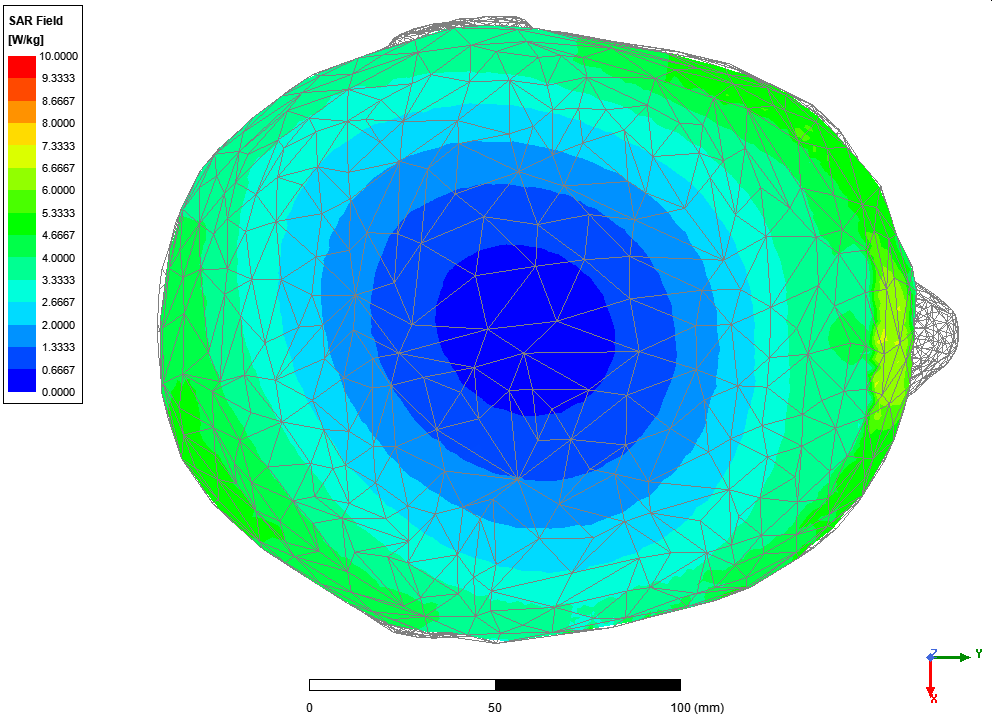


**Figure S2**: *10g Average SAR in Head Model.*


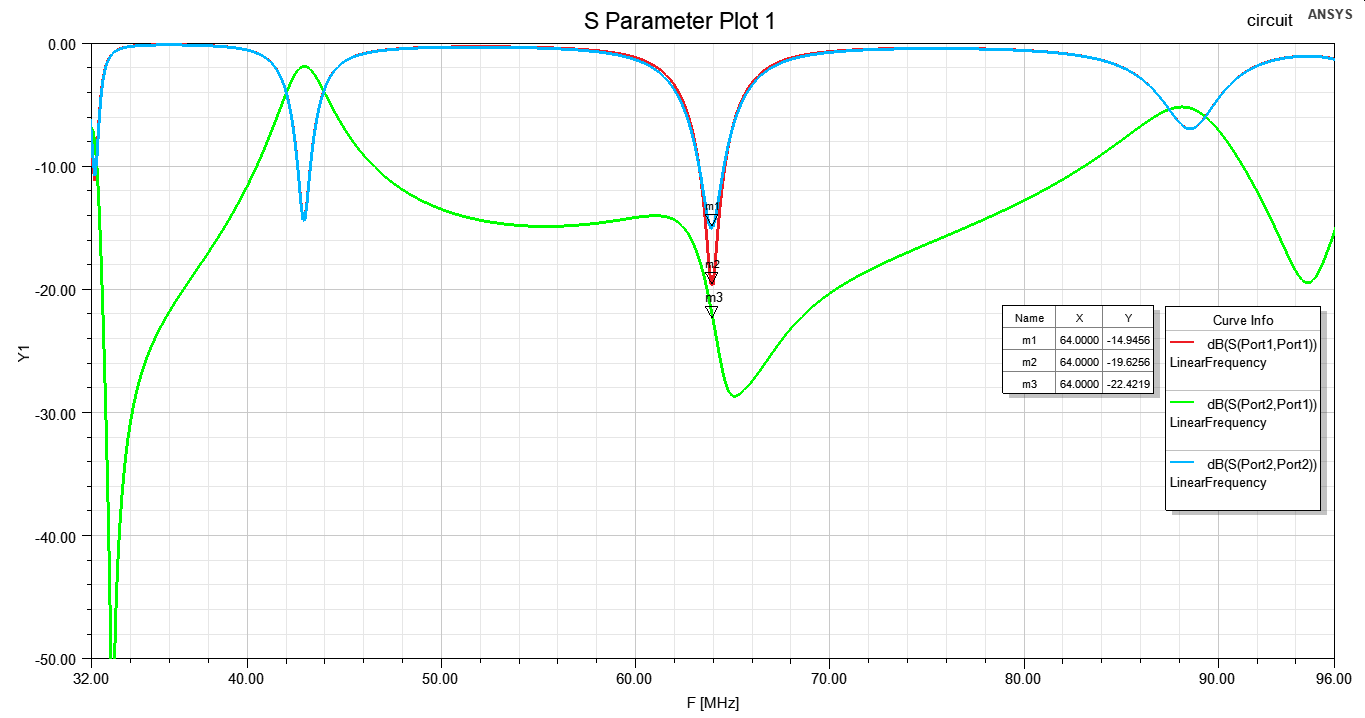


**Figure S3***: Head Coil S-Parameters.*


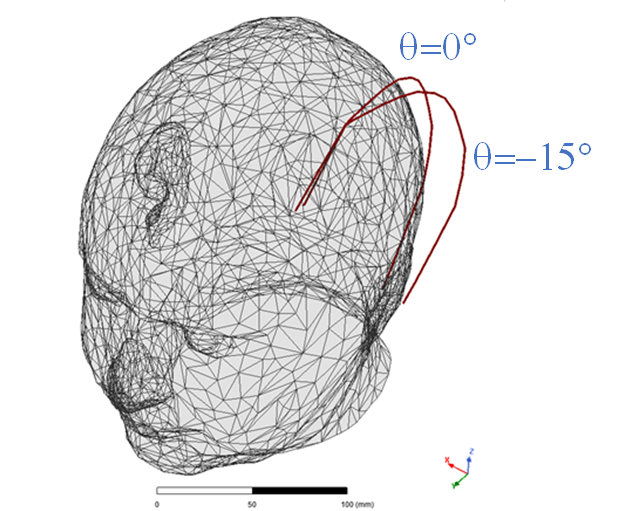


**Figure S4***: Lead Path Variation. The figure shows the two extreme angles (θ=0º and θ=-15º) of the path variation temperature study in* ***Table S2.***
